# Machine‐Learning‐Enhanced Printed Vertical Magnetoresistive Sensors for Transparent, Flexible, Multimodal Interactive Magnetoelectronics

**DOI:** 10.1002/advs.76052

**Published:** 2026-06-11

**Authors:** Rui Xu, Guannan Mu, Oleksandr V. Pylypovskyi, Qihao Zhang, Rico Illing, René Hübner, Ran He, Andreas Knüpfer, Sebastian Lehmann, Olha Bezsmertna, Kornelius Nielsch, Denys Makarov

**Affiliations:** ^1^ Institute of Ion Beam Physics and Materials Research Helmholtz‐Zentrum Dresden‐Rossendorf e.V. Dresden Germany; ^2^ Institute of Functional Materials Donghua University Shanghai China; ^3^ Institute For Metallic Materials Leibniz Institute for Solid State and Materials Research Dresden Germany; ^4^ CASUS – Center for Advanced Systems Understanding Helmholtz‐Zentrum Dresden‐Rossendorf e.V. Görlitz Germany; ^5^ Technische Universität Dresden Institute of Materials Science Dresden Germany

**Keywords:** additive fabrication, flexible electronics, human‐machine interface, machine learning, magnetoresistive sensor, transparent electronics

## Abstract

To meet the increasingly stringent demands of next‐generation electronic systems, magnetoresistive sensors are required to simultaneously deliver environmental compatibility, advanced functionality, and enhanced intelligence. Here, we demonstrate a synergistic strategy spanning device, algorithm, and system levels to address these challenges in a unified manner. By rationally designing functional inks, fully printable magnetoresistive sensors are realized through additive manufacturing, substantially reducing energy consumption and material waste during fabrication. Introducing magnetic‐field guidance during printing enables vertical alignment of functional nanowires, resulting in an out‐of‐plane sensor architecture. This configuration not only reduces nanowire surface coverage, imparting exceptional optical transparency, but also suppresses the adverse influence of inter‐nanowire junctions on electrical percolation, thereby enhancing mechanical robustness. Beyond materials and device engineering, the integration of machine‐learning algorithms and system‐level optimization extends sensor operation beyond conventional threshold‐based mechanisms, enabling robust multi‐pattern recognition capabilities. Notably, this functionality is achieved using a single sensing element without relying on sensor matrices or additional electronic components, thus preserving the intrinsic transparency and mechanical flexibility of the system. Leveraging the synergistic combination of these achievements, the proposed sensors offer an eco‐responsible platform for next‐generation imperceptible and intelligent magnetic sensing.

## Graphical abstract

This study presents printed magnetoresistive sensors with a vertically aligned architecture that enables high optical transparency and mechanical flexibility. By integrating deep learning for the analysis of complex spatiotemporal signal patterns, the system further achieves intelligent multimodal interaction capabilities. Collectively, this work establishes a sustainable and high‐dimensional perceptual platform for next‐generation interactive magnetoelectronics.

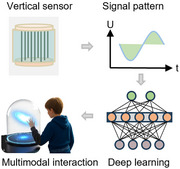

## 1 Introduction

Magnetoresistive sensors have become indispensable components in modern electronics [1, 2, 3, 4, 5], driven by the rapid proliferation of wearable devices [6, 7, 8, 9], smart healthcare systems [10, 11], transparent electronics [12, 13], virtual and augmented reality [14], and soft robotics [15, 16, 17, 18], etc. As these technologies advance, they impose increasingly stringent requirements on sensor performance, including mechanical compliance, optical transparency, and system‐level intelligence. In parallel, the mass production of such devices raises pressing environmental concerns [19], highlighting the need for sustainable innovations that can simultaneously enhance functionality and reduce ecological impact.

Substantial efforts have been undertaken to addressing these challenges. Traditional thin‐film techniques have enabled flexible and transparent magnetoresistive devices through carefully engineered layouts [20]. However, this approach relies heavily on high‐vacuum sputtering and subtractive patterning, entailing significant energy consumption and material waste. In contrast, printing technologies offer an energy‐efficient and additive alternative [21, 22, 23, 24]. Recent advancements in printed magnetoresistive sensors have been primarily realized in the form of planar composite [25, 26, 27, 28, 29, 30, 31, 32, 33]. To achieve transparency, magnetoresistive nanowires have been arranged into mesh‐like networks [34, 35], yet these conventional random networks often face a transparency‐conductivity bottleneck due to high percolation thresholds [36, 37]. Further, percolation networks involving numerous inter‐nanowire junctions are prone to degradation under continuous mechanical deformation, thereby constraining long‐term reliability. These limitations underscore the urgent need for printing strategies that decouple electrical conduction from network percolation, enabling continuous enhancement of both transparency and flexibility.

Beyond material and device considerations, the complexity of real‐world interactions poses additional challenges. Conventional magnetoresistive sensors predominantly operate through threshold‐triggered mechanisms, which are inherently inadequate for capturing rich spatiotemporal features. To overcome this, sensor arrays have been employed to exploit synergistic interactions among multiple devices. Nevertheless, such systems necessitate extensive supporting components (e.g., transistors, capacitors, and electrodes for active matrices), which inevitably increases fabrication complexity, energy consumption, and compromises both flexibility and optical clarity [38, 39]. Recently, machine learning algorithms have been employed to enhance the spatial sensing resolution of magnetic field sensors [40, 41] or to correlate simple pulse sequences for instruction generation [42]. Furthermore, machine learning algorithms have been integrated with flexible sensors to achieve high‐accuracy recognition of complex spatiotemporal pattern language and real‐time information encoding [43, 44, 45, 46]. These advancements underscore the potential of data‐driven approaches in decyphering multi‐dimensional sensing signals. Despite these advances, the reliable analysis of complex signal patterns remains a formidable challenge in magnetic interaction applications, indicating that holistic, system‐level optimization is essential for realizing intelligent magnetic sensing platforms.

In this work, we present an integrated strategy that addresses these challenges through synergistic engineering at the device, algorithmic, and system levels. By applying a guiding magnetic field during printing, we fabricate magnetoresistive sensors with vertically aligned nanowires. Unlike conventional random planar networks where nanowires must overlap extensively to reach the percolation threshold, the vertically aligned nanowire architecture creates direct, out‐of‐plane conductive channels. This transition from ‘network‐based’ to ‘column‐based’ conduction decouples electrical performance from surface coverage. Complementing materials and device design, the incorporation of machine learning algorithms and system‐level optimization enables robust multi‐pattern recognition capabilities. Beyond conventional threshold‐based operation. Importantly, this is achieved using a single sensor without introducing additional matrix circuitry, thereby preserving both transparency and flexibility. Leveraging these combined advantages, the resulting sensing system demonstrates strong potentials for applications in Internet of Things (IoT), wearable electronics, and human‐machine interfaces.

## 2 Results and Discussion

**FIGURE 1:**
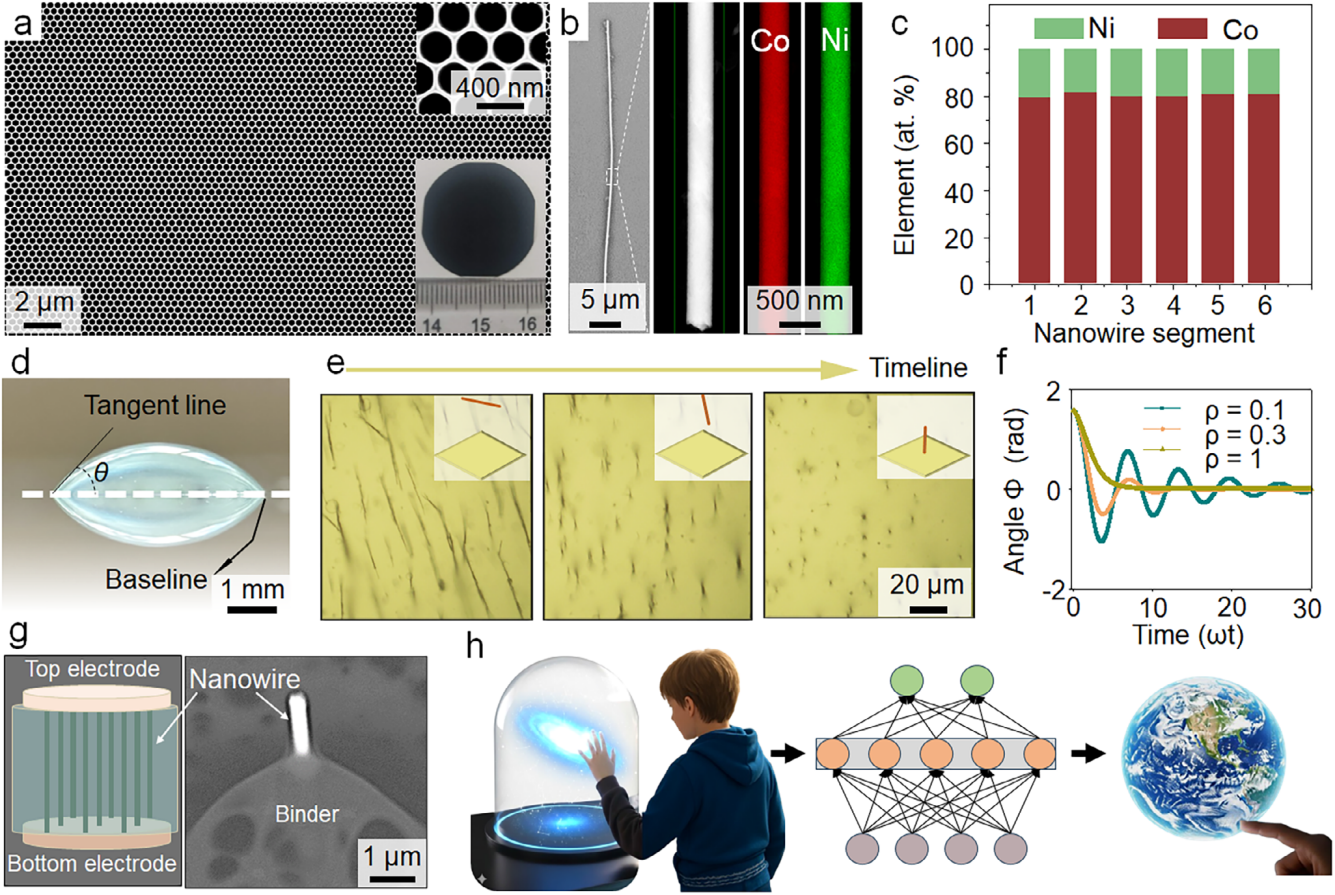
Printed magnetoresistive sensors featuring vertically oriented nanowire architecture. (a) Scanning electron microscopy (SEM) image of the nanoporous template used for magnetic nanowire fabrication, exhibiting highly ordered pores over large areas. Insets show a magnified view of the template (top) and an optical photograph of the centimeter‐scale template (bottom). (b) Representative nanowire and corresponding energy‐dispersive x‐ray (EDX) analysis of a selected segment. (c) Statistical evaluation of Co and Ni elemental ratios across six nanowires. Both analyses consistently validate compositional uniformity. (d) Contact angle measurement of the ink printed on polyethylene terephthalate (PET) substrate, indicating favorable ink wettability. (e) Optical microscopy images, showing the alignment process of nanowires dispersed in ink under a vertical magnetic field. From left to right: random dispersion before field application; partial alignment at initial field exposure; vertically aligned nanowires after stabilization. Insets depict schematic illustrations of the corresponding nanowire configurations. (f) Angular variation of nanowire orientation in inks with different viscosities under magnetic fields. A larger ρ value indicates higher medium viscosity. (g) Schematic illustration and SEM image of a vertically aligned CoNi nanowire within the composite. (h) Proof‐of‐concept intelligent interactive system. From left to right: a transparent and compliant interactive system for astronomy education. Through magnetic interactions between sensing system and user, electrical output patterns are generated; these signals are subsequently analyzed and trained within a machine learning framework; by recognizing specific interactive trajectories, the system acquires the capability to input complex commands into the virtual environment.

The realization of vertically configured printed sensors requires meticulous control over both ink formulation and printing parameters. To minimize the influence of geometric irregularities and compositional fluctuations of the nanowires on magnetoresistance behavior, a template‐assisted electrochemical deposition method is employed to synthesize nanowires with well‐controlled morphology (Figure S1) [47, 48]. The resulting nanowires feature excellent geometric uniformity, as evidenced by consistent diameters and lengths across large populations (Figure 1a, Figure S2). Energy‐dispersive x‐ray (EDX) spectroscopy confirms the homogeneous distribution of Co and Ni elements without detectable phase segregation (Figure 1b, Figure S2), while quantitative compositional analyses reveal identical element ratios (Co:Ni = 80:20) among individual nanowires (Figure 1c). This Co‐rich composition was selected based on prior studies reporting favorable magnetic anisotropy and stable nanowire magnetic behavior, making it a representative material platform for magnetoresistive sensing applications [49, 50]. To achieve an optimal balance between enhanced magnetotransport performance and the mechanical rigidity required for high‐fidelity vertical printing, nanowires with larger diameters (here, ∼ 380 nm) were exploited as functional fillers (Figure S3).

To enable vertical assembly during printing, a carefully engineered binder system was developed. Polyvinyl alcohol (PVA) was dissolved in deionized water at a mass ratio of 1:15, yielding a binder solution that undergoes volumetric shrinkage exceeding 95% upon drying. This pronounced shrinkage effectively exposes the upper ends of nanowires, facilitating reliable electrical contact formation. The choice of specialized PVA (high molecular weight, ∼ 89 000–98 000) is pivotal, because extended polymer chains confer desirable rheological properties and inhibit excessive spreading at high water contents (Figure 1b). As a result, droplet integrity is preserved during printing, providing spatial confinement that allows nanowires sufficient rotational freedom to reorient under external stimuli. Surface oxidation of metallic nanowires represents another critical bottleneck, as native oxide layers severely degrade electrical conductivity [51, 52]. To mitigate this issue, a wet‐chemical etching strategy was employed, a technique has been widely adopted in printed electronics to prevent filler oxidation [53, 54]. Following acidic treatment with phosphoric acid, the device resistance exhibited a dramatic reduction from the MΩ to the Ω scale (Figure S4). This substantial enhancement in conductivity confirms the successful establishment of robust electrical pathways. During printing, external magnetic fields (about 300 mT) are applied to guide nanowire orientation [55, 56], transforming initially random dispersions into vertically aligned assemblies (Figure 1e). Numerical simulations further suggest that, independent of ink rheology, nanowires converge to a common equilibrium orientation under magnetic guidance (Figure 1f), thereby broadening the design space for ink formulation.

The vertical sensor architecture offers distinct functionalities by circumventing the percolation threshold limitations inherent to conventional printed conductive networks (Figure 1g), thereby enabling ultra‐high transparency and mechanical flexibility, as fully validated in Figure 3. Importantly, when integrated with machine‐learning algorithms, the vertical sensing framework evolves beyond passive signal transduction and establishes an intelligent, imperceptible interaction platform, holding strong potential for next‐generation smart environments in which sensing elements operate unobtrusively while delivering advanced interactive functionalities (Figure 1h).

**FIGURE 2:**
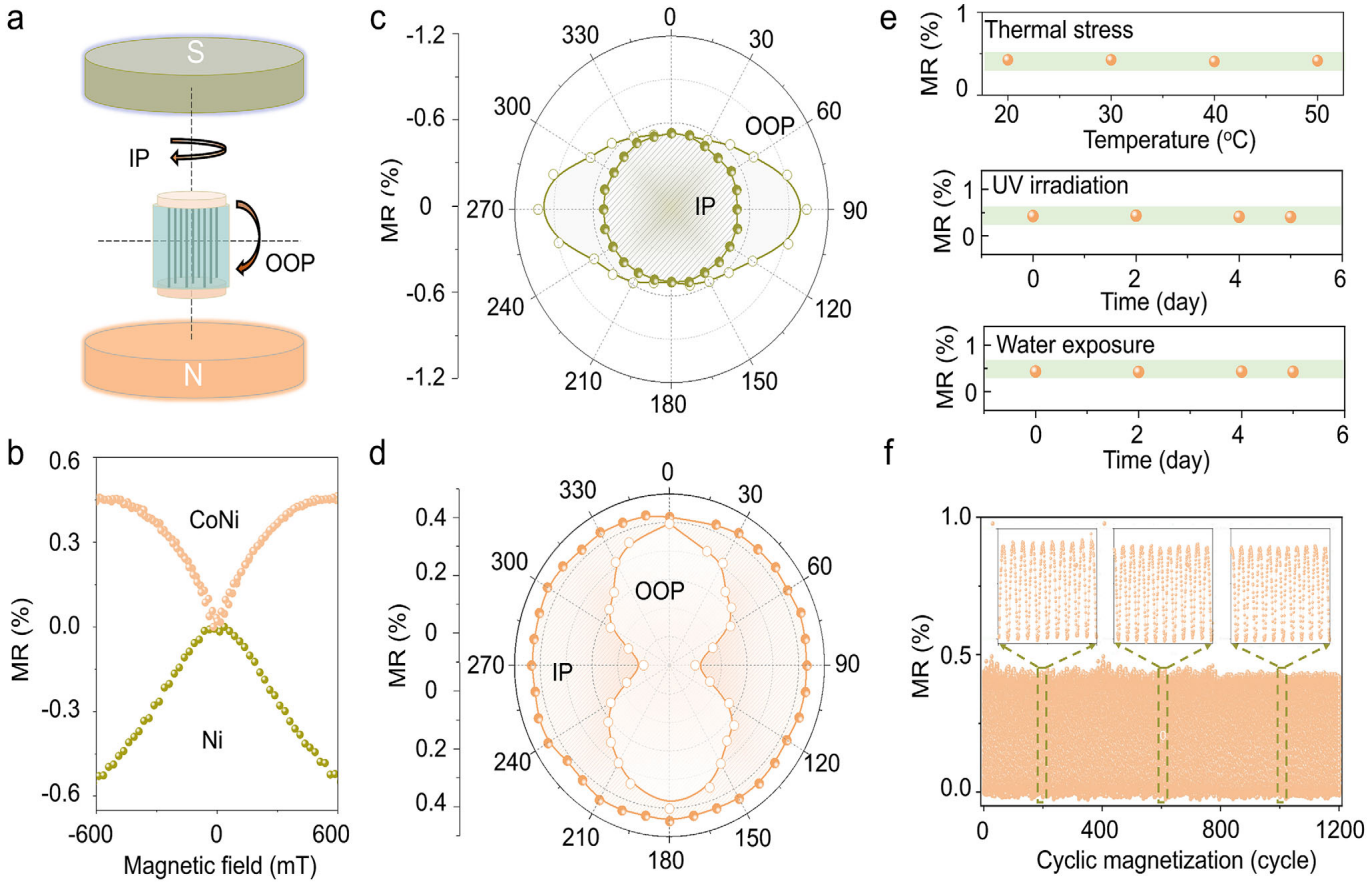
Magnetoresistance (MR) characterization of printed sensors. (a) Schematic illustration of the measurement setup, including in‐plane (IP) and out‐of‐plane (OOP) magnetic field configurations. (b) Magnetoresistance responses of sensors composed of vertically aligned Ni nanowires and CoNi nanowires. (c, d) 3D angular‐dependent magnetoresistance characteristics for (c) Ni‐based and (d) CoNi‐based sensors, obtained by rotating the sensor substrate either in‐plane (IP) or out‐of‐plane (OOP) relative to the fixed measurement setup. (e) Operational stability of CoNi‐based sensors evaluated under environmental stress, including elevated temperatures, ultraviolet (UV) radiation, and water exposure. (f) Real‐time magnetoresistance response under dynamic magnetization. Insets display ten consecutive magnetoresistance cycles at different modulation periods.

The magnetic‐field‐guided alignment strategy is not chemically specific, thus offering broad compatibility with nanowires of different compositions, ranging from elemental ferromagnetic metals to complex alloys. Measured in magnetic fields applied parallel to the nanowire axis (Figure 2a), Ni‐based sensors exhibit a negative magnetoresistance, whereas CoNi‐based devices exhibit a positive response (Figure 2b), underscoring the importance of composition modulation in determining magnetotransport behavior. To further examine orientation‐dependent effects, the sensors were subjected to controlled angular rotations. During in‐plane rotation (Figure 2a), minimal magnetoresistance variation is observed for both Ni and CoNi samples (Figure 2c,d). In contrast, out‐of‐plane rotation induces pronounced magnetoresistance anisotropy (Figure 2c,d), highlighting the dominant influence of nanowire orientation relative to the applied magnetic field. Notably, Ni‐based sensors exhibit their maximum magnetoresistance ratios when the field is perpendicular to the nanowire axis (Figure 2c), indicative of remanent magnetization preferentially aligned along the axis due to dominant shape anisotropy. In contrast, CoNi‐based sensors exhibit a distinct angular dependence, with the highest magnetoresistance observed when the magnetic field is applied approximately parallel to the nanowire axis (Figure 2d). The observed dumbbell‐like out‐of‐plane response suggests that the effective anisotropy is influenced by a competition between shape anisotropy along the nanowire axis and magnetocrystalline anisotropy associated with the hcp Co phase. This behavior is consistent with a slightly misaligned effective easy‐axis and a non‐uniform magnetization rotation process, as reported in similar CoNi nanostructures [49, 50, 57]. Importantly, by changing the direction of the guiding magnetic field during printing, nanowire orientation can be adjusted from vertical to slightly or even highly tilted configurations (Figure S5). The interplay of tunable nanowire orientation and angle‐dependent magnetoresistance behaviors thus offers a facile strategy for modulating sensor structure and performance to satisfy specific operational requirements.

To assess operational stability, the sensors are subjected to a serious of controlled stress tests. The thermal stability of the sensor was evaluated from 20°C to 50°C. This range was selected to encompass the typical operating temperatures for wearable and interactive electronics. At elevated temperatures, the magnetoresistance ratios exhibit little variation (Figure 2e, Figure S6a). The thermal resilience is primarily attributed to the PVA binder. Specifically, its glass transition, typically in the range of 75°C–85°C, allows the sensor to remain thermally stable across temperatures up to 50°C. Considering the intrinsic water solubility of the PVA binder, a waterproof layer is introduced to enhance the sensor durability in humid conditions, for example, polydimethylsiloxane (PDMS) or polymethyl methacrylate (PMMA). Encapsulated sensors maintain intact even after prolonged immersion in water (Figure 2e, Figure S6b). In addition, continuous illumination including ultraviolet (UV) radiation does not cause any noticeable decline in magnetoresistance (Figure 2e, Figure S6c). Under repeated magnetic stimulation, the sensors exhibit reproducible magnetoresistance responses without observable signal degradation (Figure 2f). Collectively, these results demonstrate the exceptional operational robustness of the vertically aligned, printed magnetoresistive sensors across a wide range of environmental and functional conditions.

**FIGURE 3:**
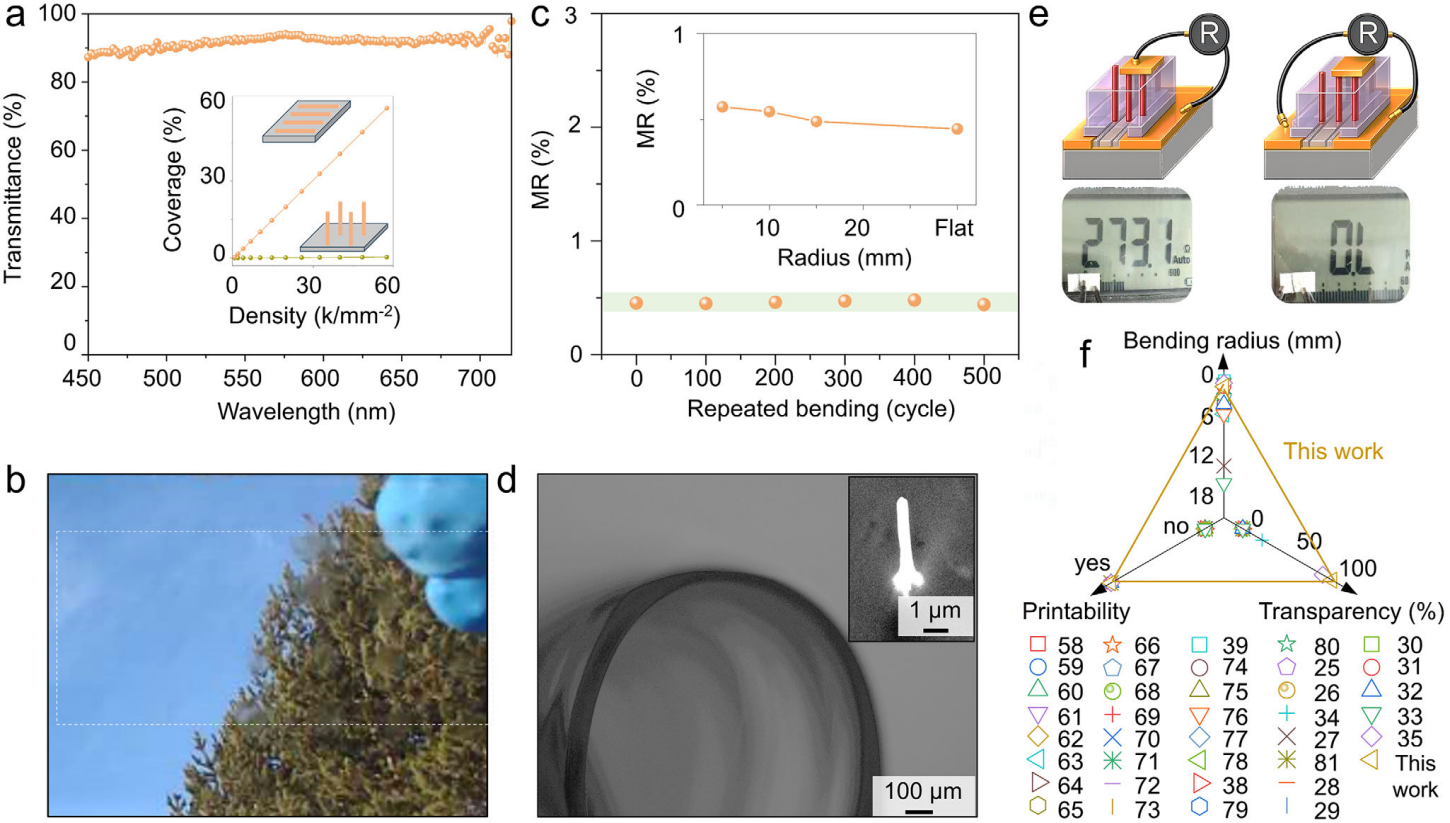
Unique properties of printed sensors enabled by the vertical sensor configuration. (a) Optical transmittance spectra of printed composite on PET in the visible range. The measurements were performed at normal incidence with air as the reference. Inset calculates the surface coverage of nanowire arrays for vertical versus lay‐down configurations. (b) Optical photograph of magnetoresistive composites printed on a transparent ITO‐sputtered glass substrate. (c) Magnetoresistance (MR) ratios under cyclic bending. Insets records magnetoresistance variation as a function of bending radius. Silver paste electrodes are employed to accommodate mechanical bending, considering the rigidity of the ITO‐coated substrate. (d) Optical microscopic image of the magnetic composite under bending. Inset shows the corresponding SEM image, confirming no interfacial separation between binder and nanowire under bending. (e) Vertical sensor architecture, mitigating lateral electrical shorting. Top: schematic diagrams of the measurement configuration. Bottom: corresponding measured resistance values. (f) Comparative analysis of previously reported unconventional sensors (e.g., flexible or printable sensors) and the vertical sensor [25, 26, 27, 28, 29, 30, 31, 32, 33, 35, 38, 39, 58, 59, 60, 61, 62, 63, 64, 65, 66, 67, 68, 69, 70, 71, 72, 73, 74, 75, 76, 77, 78, 79, 80, 81], highlighting three key functional aspects: mechanical flexibility, optical transparency, and printable (i.e., energy‐ and material‐efficient) manufacturing.

Benefiting from the vertical configuration, the printed sensors gain a set of extraordinary properties. Most notably, the vertical alignment overcomes the percolation threshold constraint that typically governs electrical transport in composite‐based sensors (Figure S7). The vertical architecture achieves reliable conduction even with a surface coverage below 1% (Figure 3a). This drastic reduction in nanowire footprint effectively suppresses light scattering, resulting in ultrahigh optical transparency. As shown in Figure 3a, the magnetoresistive composite printed on a PET foil exhibits optical transmittance exceeding 90% across the entire visible spectrum. Notably, the vertically aligned architecture decouples the device performance from the filler concentration, as the magnetotransport properties are predominantly governed by the intrinsic characteristics of individual nanowires rather than the density of the lateral percolation network. Consequently, the sensor exhibits highly consistent magnetoresistance performance across a broad range of loading fractions (Figure S8). In addition, this vertical conduction pathway fundamentally alters the mechanical failure mode of the device. Considering the intrinsic rigidity of transparent ITO electrodes, silver paste electrodes are employed to establish compliant electrical contacts. As shown in Figure 3c, the sensors maintain stable magnetoresistive responses under bending to curvature radii as small as 5 mm and exhibit negligible performance degradation after 500 bending/unbending cycles. Even under extreme deformation, the nanowires remain firmly embedded within the polymer binder without observable interfacial delamination, ensuring structural integrity of the composite. The mechanical robustness stems from its unique vertical conduction architecture. In conventional percolation theory, the electrical path is a series‐parallel network of junctions (Figure S9). These junctions are the weakest links under mechanical load. By contrast, the magnetic‐field‐induced vertical alignment creates quasi‐independent conductive pillars. Bending substrate has a minimal effect on the out‐of‐plane resistance, unlike planar networks where nanowire junctions are easily loosened. The ultimate bending tolerance of the device is therefore expected to be governed by the reliability of electrical contacts between the vertically aligned nanowires and the top and bottom electrodes, highlighting future interface optimization. In addition to optical and mechanical advantages, the vertical conduction channels intrinsically minimize the risk of lateral electrical short‐circuiting that commonly plagues planar interconnects (Figure 3e). Benchmarking against previously reported magnetoresistive sensors reveals that the present vertical sensors achieve either the highest transparency or among the best mechanical flexibility, thereby enabling application scenarios that are largely inaccessible to rigid or optically opaque counterparts [25, 26, 27, 28, 29, 30, 31, 32, 33, 35, 38, 39, 58, 59, 60, 61, 62, 63, 64, 65, 66, 67, 68, 69, 70, 71, 72, 73, 74, 75, 76, 77, 78, 79, 80, 81]. Importantly, these unique properties are realized through material‐ and energy‐efficient printing processes, positioning the vertical sensors as promising building blocks for future sustainable electronic systems.

**FIGURE 4:**
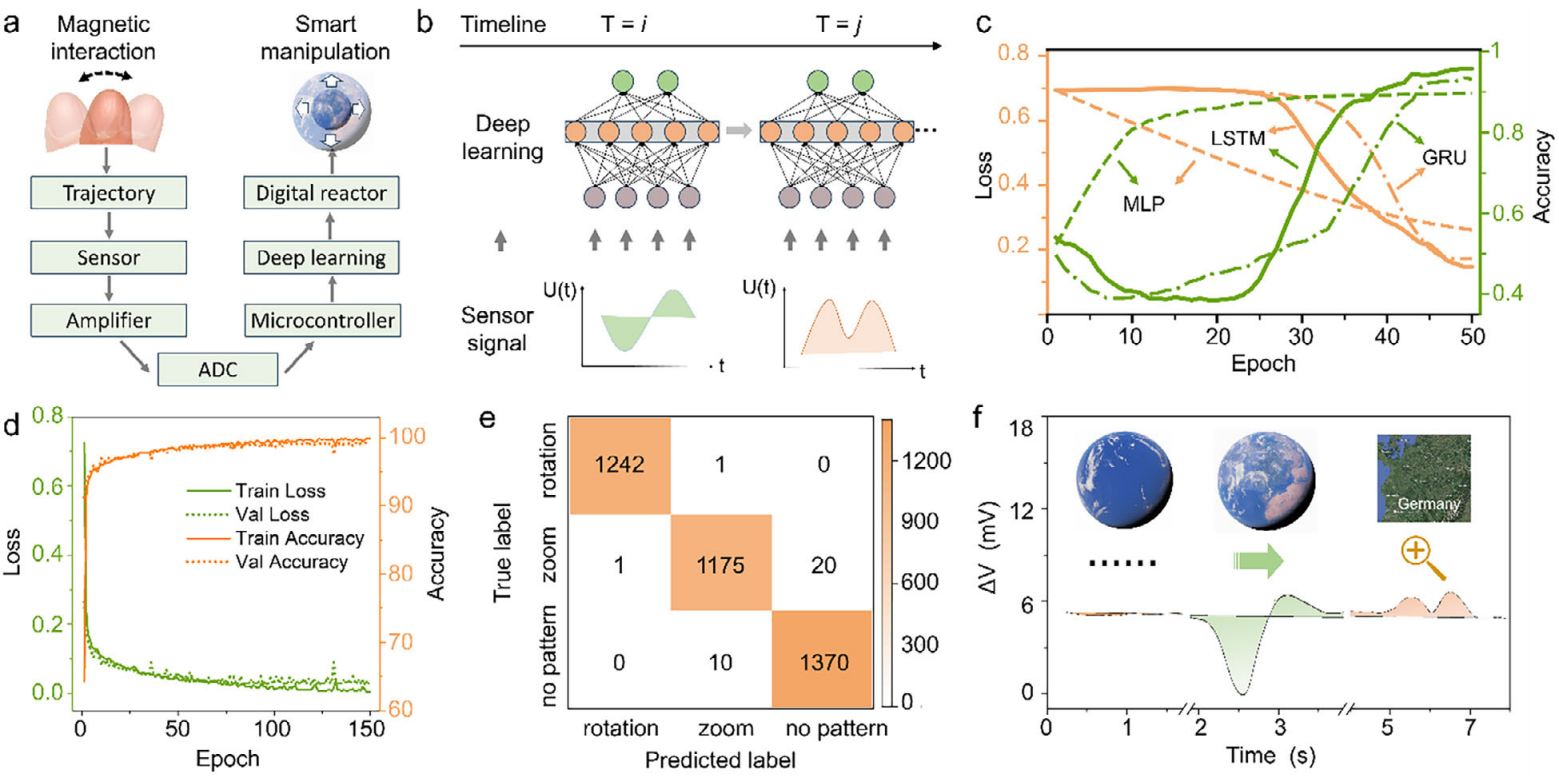
Machine‐learning‐augmented human‐machine interaction enabled by on‐skin magnetoresistive sensors. (a) Workflow of the magnetic sensing system integrated with machine learning for time‐series pattern recognition. (b) Conceptual illustration of machine‐learning‐based time‐series pattern recognition. (c) Mean validation loss and accuracy curves of multilayer perceptron (MLP), Gated Recurrent Unit (GRU), and Long Short‐Term Memory (LSTM) over ten independent training runs. Please refer to Table S1 for model parameters. (d) Training performance of the LSTM model for time‐series pattern recognition. Training data augmentation is used to generate larger datasets that keep the characteristics of the manually measured signals of each category, see Figure S13 and Table S2. (e) Evaluation of the LSTM classification using a confusion matrix (rotation, zoom, and no‐pattern). (f) Experimental demonstration of digital control based on temporal patterns, e.g., cosine‐like and bimodal waveforms.

To enable intelligent interpretation of complex spatiotemporal magnetic signals without compromising transparency or flexibility, a compact machine‐learning framework is implemented using a single sensor, as illustrated in Figure 4a,b. The “intelligence” of the proposed sensor system is defined as its capability to interpret complex, time‐varying magnetic signals that are otherwise indistinguishable using conventional peak‐detection or threshold‐based methods. Specifically, the system utilizes learning models to achieve high‐accuracy temporal pattern classification. During operation, voltage signals generated by the sensor in response to finger movements are continuously sampled and streamed to the microcontroller. The acquired voltage signals are preprocessed and partitioned into separate datasets for training, validation, and deployment of machine learning model (Figure S10).

To identify a suitable neural network architecture for signal pattern analysis, first, using the same lightweight dataset, we evaluate and compare multilayer perceptron (MLP), Long Short‐Term Memory (LSTM), and Gated Recurrent Unit (GRU) neural networks, as each one offers distinct features in terms of complexity, temporal modeling capacity, and computational efficiency [82, 83]. Figure 4c summarizes the averaged validation loss and accuracy curves of the three neural network architectures over ten independent training runs, where all three models show convergence behavior but reached different final validation performances. Leveraging global shape‐level features from the input sequence, the MLP network delivers fast training while achieving an average final validation accuracy of 89.8% ± 1.8% (Figure S11). However, due to the lack of temporal information flow between input time steps, it is not well‐suited for capturing temporal dependencies inherent in sequential data. This limitation is anticipated to become more pronounced in future applications involving a larger number of categories and more complex temporal dynamics. Recurrent Neural Network (RNN) with intrinsic memory capabilities and some of their variants could address this limitation. For example, by capturing dynamic temporal dependencies through its input, forget, and output gates, LSTM enables stronger temporal feature extraction and achieves a high average final validation accuracy of 95.8% ± 1.7% (Figure S11). In contrast to LSTM, another recurrent neural network, GRU, employs a streamlined architecture and achieved a slightly lower average final validation accuracy of 93.3% ± 2.7% (Figure S11). Beyond classification accuracy and training stability, inference latency and scalability to more complex tasks were further evaluated for model comparison. Although MLP showed the shortest model‐level inference latency of 0.060 ± 0.011 ms, LSTM still retained a low forward‐pass latency of 0.322 ± 0.038 ms, which was substantially lower than that of GRU under the same implementation conditions (0.899 ± 0.077 ms) and suitable for real‐time signal classification (Figure S12). Moreover, the scalability of the three models was evaluated by progressively increasing the classification complexity from binary recognition between no‐pattern and rotation (a static and a dynamic pattern), to binary recognition between rotation and zoom (two distinct dynamic patterns), and finally to three‐class recognition of no‐pattern, rotation, and zoom (Table S3). With increasing task complexity, MLP and GRU showed accuracy decreases from 95.0% to 86.7% and from 97.5% to 90.0%, respectively, whereas LSTM showed a smaller decrease from 97.5% to 93.3% and maintained the highest accuracy in the three‐class task. These results demonstrate better scalability of LSTM to more complex temporal pattern‐classification tasks. Therefore, LSTM was selected as the final neural network architecture for subsequent deployment and further experimentation because it provided the best balance among classification accuracy, training stability, inference latency, and scalability to more complex temporal pattern‐recognition tasks.

Figure 4d presents the complete training and validation curves of loss and accuracy for the LSTM model over 150 epochs. Both training and validation losses drop steeply during the first five to ten epochs, accompanied by a rapid increase in accuracy from about 60% to >95%. Subsequent improvements proceed more gradually until about 30–40 epochs, after which all curves reach a plateau. By the end of training (150 epochs), the model stabilizes at 99.8% training accuracy and 98.8% validation accuracy, with losses approaching zero. The close alignment of the training and validation curves, with a nearly constant generalization gap of about 1% and only minor stochastic fluctuations in the validation loss, indicate good generalization and no overfitting. Consistent with these metrics, the confusion matrix on the independent test set (Figure 4e) indicates uniformly high per‐class performance: rotation 1242/1243 (99.9%), zoom 1175/1196 (98.2%), and no‐pattern 1370/1370 (100%). Residual errors occur primarily from zoom to no‐pattern (20 samples), with negligible cross‐confusion otherwise. In practical scenarios, incoming voltage sequences are processed and classified in real‐time into one of several learned spatiotemporal patterns. As a representative use case, different motion patterns are mapped to predefined controls. For instance, cosine‐like and bimodal signals are respectively interpreted as zoom‐in and rotating commands on a digital Earth visualization platform (Figure 4f).

## 3 Conclusion

In summary, this work establishes a new paradigm for magnetoresistive sensors by holistically integrating device architecture, sustainable manufacturing, unique functionalities, and system‐level intelligence. Leveraging additive printing processes, the proposed approach substantially reduces material consumption and energy demand, thereby minimizing the environmental footprint of sensor fabrication. Through carefully engineered ink formulation combined with magnetic‐field‐guided assembly, vertically aligned nanowires are realized within printed composites. In stark contrast to conventional lay‐down architectures, this vertical configuration enables a unique combination of ultrahigh optical transparency and mechanical flexibility. At the system level, integration with machine learning frameworks transcends traditional threshold‐based sensing, enabling the extraction and interpretation of complex spatiotemporal electrical signal patterns. While the current iteration does not yet feature real‐time adaptation or context‐aware reasoning, it demonstrates a significant step toward moving beyond binary sensing to sophisticated spatiotemporal pattern interpretation. Importantly, this intelligence is based on a single sensor element without resorting to active matrix and supporting electronics, thereby opening a pathway toward intelligent interaction modalities without compromising optical invisibility or mechanical compliance. Collectively, these advances lay the foundation for next‐generation magnetoresistive sensing platforms that are sustainable, imperceptible, and intelligent, with broad implications for ubiquitous sensing technologies.

## 4 Experimental Section

### 4.1 Fabrication of Magnetoresistive Nanowires

As depicted in Figure S1, the fabrication of nanowires was carried out using a template‐assisted method [68, 69, 75]. Anodic aluminum oxide (AAO) templates were prepared via the anodic oxidation of high‐purity aluminum foils. Initially, a custom‐fabricated nickel stamp with an ordered nanopillar array was employed to imprint electropolished aluminum foils through mechanical indentation, producing periodic surface nanodents with a pitch of 400 nm. The patterned aluminum foils were then subjected to anodization in a 5 wt.% phosphoric acid solution under a constant voltage of 160 V. The resulting pore length was modulated by the anodization time, ranging from several tens of nanometers to several hundred micrometers, while the pore diameter was tuned from 120 to 380 nm via wet‐chemical etching. Subsequently, thin layers of chromium (10 nm) and gold (20 nm) were sequentially deposited onto the anodized surface, followed by the electrodeposition of a thick copper layer. The copper layer functioned both as a mechanical support for the rigid, nonporous template and as a conductive base electrode for subsequent electrodeposition. The remaining Al substrate was completely removed by immersion in a mixed solution of hydrochloric acid and copper(II) chloride. To expose the copper layer and enable nanowire growth, the AAO templates were further treated in diluted H_3_PO_4_ to open the pore bottoms. To improve compositional uniformity, particularly for alloy nanowires, a three‐electrode electrochemical configuration was employed. This setup allows for precise potential control, which is critical for co‐depositing elements with different standard reduction potentials and for suppressing local composition fluctuations during growth. Accordingly, CoNi nanowires were electrodeposited using a three‐electrode configuration, with an Ag/AgCl reference electrode and a Pt mesh counter electrode. The electrolyte consisted of an aqueous solution containing 0.2 m cobalt sulfate, 0.95 m nickel sulfate, 0.16 m nickel chloride, and 0.73 m boric acid. The composition of the resulting alloy nanowires was controlled by adjusting the deposition potential, with a value of −0.9 V (vs. Ag/AgCl) yielding Co_80_Ni_20_ nanowires. In contrast, electrodeposition of pure Ni nanowires was conducted using a two‐electrode configuration. The electrolyte comprised an aqueous solution containing 0.38 m nickel sulfate, 0.12 m nickel chloride, and 0.5 m boric acid, with a nickel foil employed as the counter electrode. Finally, the templates were completely removed by immersion in 2 m sodium hydroxide solution for 4 h, and the freestanding nanowires were released from the substrate via ultrasonic agitation.

### 4.2 Printable Fabrication of Magnetoresistive Sensors

In the formulation of printable magnetic inks, the synthesized CoNi and Ni nanowires were employed as magnetoresistive fillers. The polymeric binder matrix was composed of polyvinyl alcohol (PVA) dissolved in deionized (DI) water. The sensor fabrication was carried out on glass or flexible plastic substrates, which also served as the mechanical support. The conductive electrode pads were fabricated from either ITO or commercially available silver pastes. Prior to deposition, the magnetic ink was homogenized using a commercial vortex mixer for 1 min to ensure uniform dispersion of nanowires within the ink. The prepared ink was then drop‐cast onto predefined regions of the substrate to form the active sensing elements. During the drying process, a magnetic field of approximately 500 mT, oriented perpendicular to the substrate surface, was applied until the ink was completely dried, thereby promoting vertical alignment of the nanowires through magnetic guidance. To improve electrical contact between the nanowires and the electrode pads, the insulating oxide layer naturally formed on the nanowire surfaces was removed via chemical treatment with a diluted phosphoric acid solution (0.05 m). To ensure long‐term operational stability and mitigate environmental degradation, the printed sensor architectures were encapsulated with a functional protective layer. This encapsulation step is critical given the hygroscopic nature of the PVA binder, which is susceptible to moisture and external stressors such as sweat during wearable applications. Depending on the specific functional requirements, two distinct polymer systems were utilized. For wearable demonstrations prioritizing superior mechanical flexibility and biocompatibility, PDMS (Sylgard 184, 7:1 base‐to‐curing agent ratio) was drop‐cast onto the active sensing area and thermally cured at 70°C for 1 h. For devices requiring exceptional optical clarity and higher structural rigidity, PMMA was employed as the encapsulant. The PMMA solution was prepared by dissolving the polymer in propylene glycol monomethyl ether acetate (PGMEA) at a concentration of 10 wt.%, followed by controlled deposition and curing. These dual encapsulation strategies verify the versatility of our printed sensors across diverse operating environments, ranging from flexible wearables to transparent optoelectronics.

### 4.3 Characterization of Magnetoresistive Sensors

To assess the magnetoresistive performance of the printed sensors, they were subjected to external magnetic fields, generated by an electromagnet. Electrical resistance was monitored using a Tensormeter (HZDR Innovation GmbH, Germany). The magnetoresistance (*MR*) ratios were calculated as:

$$MR=\left(R_{H}-R_{0}\right)/R_{0}$$

where *R_H_* represents the electrical resistance of the sensor measured with magnetic field *H*, and *R_0_* is the electrical resistance under zero magnetic field.

To assess the thermal stability of the printed sensors, they were placed on a hot plate. The sensors were exposed to various temperatures within the oven for 10 min before being measured at room temperature.

To evaluate the reliability of sensor performance in moisture environments, the sealed sensors were immersed in water. Every 24 h the sensors were taken out from water for magnetoresistive characterization.

To test the mechanical robustness of the printed sensors, curved sample holders with radii of 3 and 6 mm were utilized to impose mechanical bending to the sensors. Following the aforementioned method, the magnetoresistance values of the sensors were recorded.

### 4.4 Demonstrator of the Sensor

A compact temporal pattern recognition system was developed to enable machine‐learning‐assisted human‐machine interaction. The system integrated a magnetoresistive sensor, a microcontroller unit (Raspberry Pi Pico W, Raspberry Pi Foundation), and an external analog‐to‐digital converter (ADS1115, Texas Instruments), enabling real‐time acquisition and processing of motion‐induced voltage signals. To extract and classify spatiotemporal features embedded in these signals, a recurrent neural network architecture based on Long Short‐Term Memory (LSTM) was implemented. The LSTM model was selected due to its proven capability in capturing temporal dependencies within sequential data. During operation, time‐varying voltage signals were generated by the magnetoresistive sensor in response to relative motion between the magnet and the sensor. Data acquisition was performed using the Raspberry Pi Pico W, with manually controlled movement patterns ensuring consistent input conditions. Each temporal sequence was labeled according to the corresponding motion trajectory. Preprocessing included z‐score normalization of raw voltage signals to ensure uniform feature scaling, followed by reshaping the data into 3D arrays conforming to the input requirements of LSTM‐based networks. Motion labels were encoded into categorical integers suitable for supervised classification. The resulting dataset was randomly divided into training, validation, and test subsets according to a predefined ratio. The LSTM model architecture consisted of a single recurrent LSTM layer, followed by a fully connected dense layer with softmax activation for multi‐class classification. Model training was conducted using the Adam optimization algorithm with categorical cross‐entropy as the loss function. Training was performed over multiple epochs, during which model performance was continuously monitored via accuracy and loss metrics on both training and validation sets. To further improve the performance of the LSTM model, we employed a time‐series data augmentation strategy to expand the training dataset. The detailed augmentation procedure and representative examples of original and augmented signals are provided in Figure S13. In brief, additional time‐series signals were generated through pairwise linear averaging of experimentally measured signals with similar temporal characteristics, allowing the training set to include moderate intra‐class variations while preserving the class‐specific temporal profiles. To evaluate the effect of this strategy, we compared the LSTM performance with and without data augmentation on independent validation data (Figure S14). The augmented model achieved a higher validation accuracy of 98.6%, compared with 93.3% without augmentation, while the validation loss decreased from 0.223 to 0.042. These results indicate that the data augmentation strategy improves the generalization capability of the LSTM model on independent validation data. Standard classification metrics, including overall accuracy and confusion matrix were used to evaluate model performance. Training dynamics were visualized through epoch‐wise plots of loss and accuracy. Upon convergence, the trained model was quantized and exported alongside its preprocessing pipeline for deployment on the Raspberry Pi Pico W. For real‐time inference, incoming voltage sequences were processed onboard and classified into one of several predefined spatiotemporal motion categories. As a representative use case, each recognized motion pattern was mapped to a specific control command, such as zoom‐in or rotation, within a Google Earth‐based interface.

To further characterize the practical real‐time performance of the digital‐control interface, we quantified both the input‐window‐related latency and the algorithmic processing latency. Each LSTM classification used a 20‐point voltage input window sampled at an interval of approximately 0.120 s, corresponding to an input‐window temporal span of approximately 2.28 s. This complete temporal context was used to ensure that the full motion pattern was sufficiently captured for reliable classification. Assuming an approximately uniform distribution of motion‐event completion within the input window, the median effective input‐window latency was estimated to be approximately 1.14 s. In the real‐time demonstration, the input window was updated using a 5‐point sliding step, resulting in a classification evaluation interval of approximately 0.60 s and enabling repeated real‐time evaluation of incoming temporal patterns. Once the input window was available, the total algorithmic latency was only 0.660 ± 0.117 ms, corresponding to an algorithmic processing speed of approximately 1515 classifications s^−^^1^. Together, the effective input‐window latency, sliding‐window evaluation interval, and millisecond‐scale algorithmic latency support practical real‐time operation for the demonstrated virtual Earth control task and related command‐level digital HMI applications. A detailed latency breakdown is summarized in Table S4.

### 4.5 Simulation About Rotation of a Nanowire in a Magnetic Field

We consider a long magnetically soft nanowire of length *L*, radius *R* ≪ *L*, mass *m* immersed in a liquid. Initially, the wire is positioned horizontally under an angle *ϕ_0_* = 0. Being exposed to a magnetic field along the vertical direction, the nanowire rotates toward the equilibrium value of *ϕ* = π/2. Its rotational motion is governed by the equation:

(1) $$\frac{d^{2}\phi }{dt^{2}}I=\mu B\cos\phi -2r_{1}\frac{d\phi }{\mathrm{dt}},\;\phi \left(t=0\right)=\phi_{0},\;{\left.\frac{d\phi }{\mathrm{dt}}\right|}_{t=0}=0$$

Here, *I* = *mL* ^2^ / 12 is the moment of inertia of the wire, *µ* = π*R* ^2^ *LM*_0_ is the magnetic moment of the wire, *M*_0_ is the saturation magnetization, *B* is the magnetic field along z‐axis and *r_1_* is the coefficient characterizing viscous properties of the medium with respect to the rotational motion of a rigid rod. Equation (1) for the damping‐free regime (*r_1_* = 0) has the solution

(2) $$\phi \left(t\right)=\frac{\pi }{2}+2\text{am}\left(\frac{\omega_{0}t-K\left(1/2\right)}{\sqrt{2}},2\right)$$

where am(•, *k*) is the Jacobi amplitude with elliptic modulus *k*, $\omega_{0}=\sqrt{\mu B/I}$ is the characteristic frequency and K(•) is the complete elliptic integral of the first kind. For the material parameters (including *m* = 8.7 pg, *L* = 5 µm, *R* = 250 nm, *M*_0_ = 800 kA/m), the characteristic time scale T = 2π/ω_0_ ∼ 100 µs. For the case of damped motion, Equation (1) can be solved numerically. Figure 1f shows *ϕ*(*t*) for different effective viscosities.

The motion of a nanowire under the combined action of gravity and viscous forces is described by the Newton's law

(3) $$m\frac{d^{z}}{dt^{2}}=-\mathrm{mg}-2r_{2}\frac{\mathrm{dz}}{\mathrm{dt}}$$

where *z* is the vertical coordinate of the center of mass of the nanowire, *g* is the gravity acceleration, and *r_2_* is the coefficient characterizing viscous properties of the medium with respect to the linear motion of the rod. Equation 3 for the initial conditions *z*(0) = *h*_0_ and dz/dt(0) = 0 has the solution

(4) $$z\;\left(t\right)=h_{0}+\frac{g}{4r_{2}^{2}}\left(1-e^{-2r_{2}t}\right)-\frac{g}{2r_{2}}t$$

where *h_0_* is the initial height at which the nanowire is placed.

Simulation for *L* = 20 units is done for *ρ* = *r_1_* / (*ω_0_I*) = 0.1, *ρ_2_* = *r_2_* / (*ω_0_m*) = 0.01 and *g* / ω_0_^2^ = 0.0319. Time is measured in units of 1/ω_0_.

The magnetic‐field‐guided alignment is a physically universal process governed by the minimization of magnetostatic energy. For ferromagnetic nanowires made of any magnetically soft material (e.g., Ni, Co, Fe, and their alloys), a homogeneous external field induces a magnetic torque that can outweigh the rotational friction in the polymer matrix. To demonstrate this broad compatibility, we experimentally extended the strategy to Ni, NiFe, and CoNi nanowires, achieving a consistent out‐of‐plane architecture and reliable sensing performance (Figure 2, Figure S15).

### 4.6 Optical Transmittance Measurement

The optical transmittance of the printed sensors was characterized using a lab‐integrated spectrophotometer. A broad‐spectrum light source was employed to cover the visible wavelength range (400–800 nm). All measurements were conducted at normal incidence. To provide a conservative assessment of device transparency, the instrument was calibrated using air as the reference.

## Author Contributions

**Rui Xu**: conceptualization, investigation, writing – original draft, visualization, methodology, validation, writing – review and editing, formal analysis, supervision. **Guannan Mu**: investigation, writing – original draft, methodology, visualization, software, formal analysis, conceptualization, validation, writing – review and editing. **Oleksandr Pylypovskyi**: investigation, writing – original draft, methodology, visualization, writing – review and editing, formal analysis, software. **Andreas Knüpfer**: investigation, writing – original draft, writing – review and editing, methodology, software, supervision. **Olha Bezsmertna**: investigation, writing – review and editing, visualization, methodology. **Sebastian Lehmann**: investigation, writing – review and editing, methodology, visualization, formal analysis. **René Hübner**: investigation, writing – original draft, writing – review and editing, methodology, visualization, formal analysis. **Rico Illing**: investigation, writing – review and editing, methodology, formal analysis. **Qihao Zhang**: investigation, writing – review and editing, methodology, conceptualization, visualization. **Denys Makarov**: conceptualization, investigation, funding acquisition, writing – original draft, writing – review and editing, resources, supervision, project administration, visualization. **Kornelius Nielsch**: conceptualization, funding acquisition, writing – review and editing, methodology, project administration, supervision, resources. **Ran He**: investigation, writing – original draft, writing – review and editing, methodology, visualization.

## Ethics

In several studies, a sensor was applied on the skin of a user. These studies were done according to the ethic approval #SR‐EK‐459122024 from the ethics committee at the Technical University of Dresden. For this case, we have a written consent of the user (one volunteer, male, 37 years old), who was wearing this sensor. The current lines were always isolated and are not in touch with the skin. The sensor was not worn on the skin for any extended duration.

## Conflicts of Interest

The authors declare no conflicts of interest.

## Supporting information




**Supporting File 1**: advs76052‐sup‐0001‐SuppMat.pdf.

**Supporting File 2**: advs76052‐sup‐0002‐VideoS1.mp4.

## Data Availability

The data that support the findings of this study are available from the corresponding author upon reasonable request.
