# Supplementary material for: Machine‐Learning‐Enhanced Printed Vertical Magnetoresistive Sensors for Transparent, Flexible, Multimodal Interactive Magnetoelectronics
Source: Adv Sci (Weinh). 2026 Jun 11:e76052. Online ahead of print. doi: 10.1002/advs.76052 (PMC13336623; doi:10.1002/advs.76052)
Supplement: Supplementary file 1 — Supporting File 1: advs76052‐sup‐0001‐SuppMat.pdf. [file ADVS-9999-e76052-s001.pdf]

Supporting Information

Printable and recyclable vertical magnetoresistive sensors for ultra-transparent, flexible, machine learning-enabled multimodal interactive magnetoelectronics

Rui Xu,[⊥ *] *Guannan Mu,*[⊥] *Oleksandr Pylypovskyi, Qihao Zhang, Rico Illing, René Hübner, Ran He, Andreas Knüpfer, Sebastian Lehmann, Olha Bezsmertna, Kornelius Nielsch, Denys Makarov*[*]

R. X., G. M., O. P., Q. Z., R. I., Re. H., D. M.
Helmholtz-Zentrum Dresden-Rossendorf e.V., Institute of Ion Beam Physics and Materials Research, 01328 Dresden, Germany.

E-mail: r.xu@hzdr.de; d.makarov@hzdr.de
[⊥]Rui Xu and Guannan Mu contributed equally to this work.

A. K.
Helmholtz-Zentrum Dresden-Rossendorf e.V., CASUS - Center for Advanced Systems Understanding, Görlitz, Germany

Ra. H., S. L., K. N.
Institute for Metallic Materials, Leibniz Institute for Solid State and Materials Research, 01069 Dresden, Germany

K. N.
Technische Universität Dresden, Institute of Materials Science, 01062 Dresden, Germany

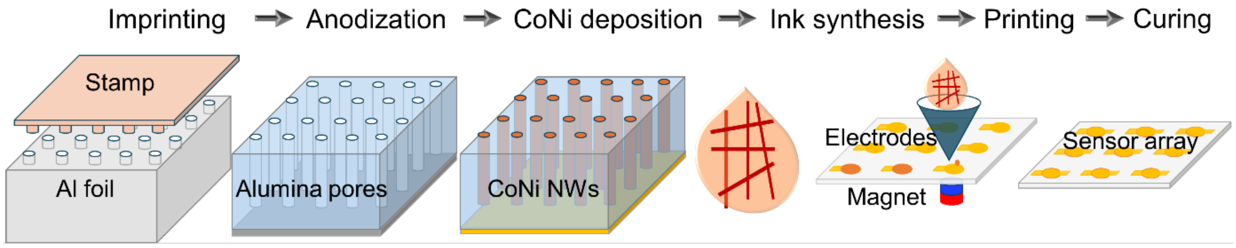

Figure S1. Template-guided synthesis of magnetoresistive (CoNi) fillers and printable fabrication of magnetoresistive sensors.

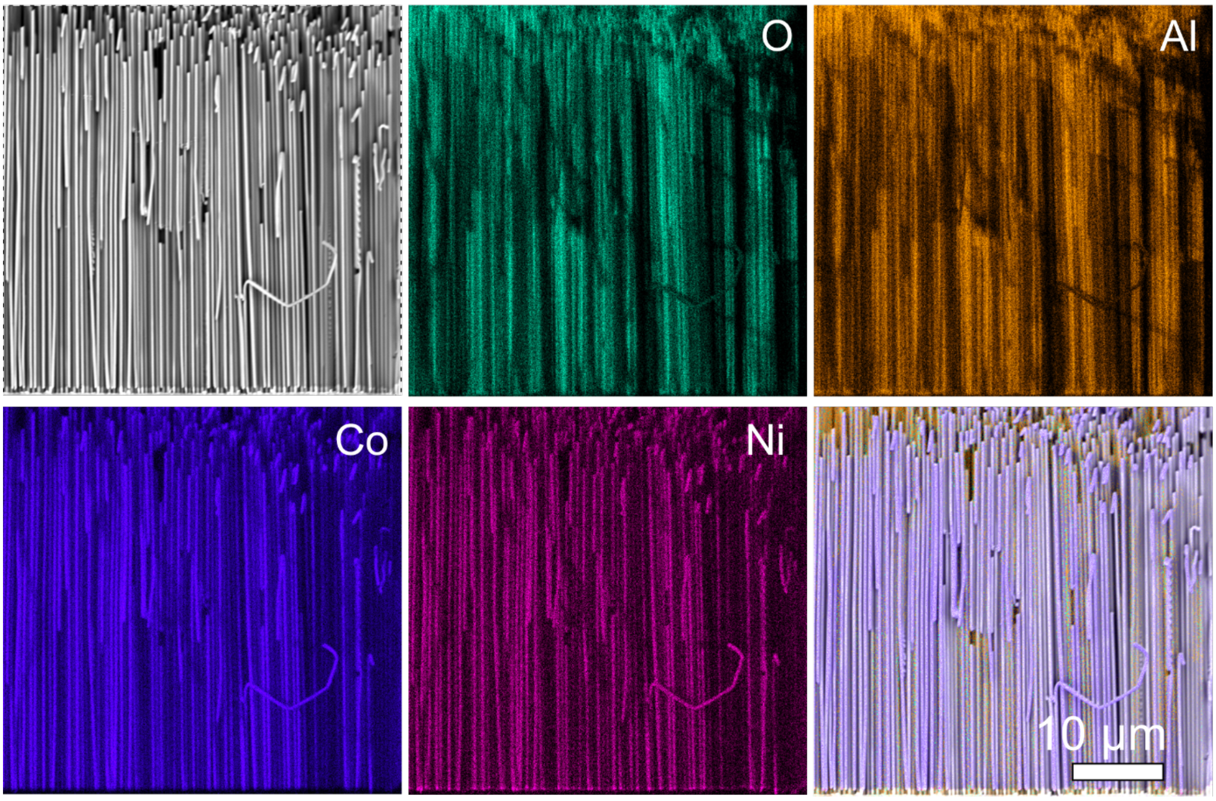

Figure S2. CoNi nanowires filled in nanoporous template. The cross-sectional SEM image accompanied by element mapping of Co, Ni, and the template-relevant elements displays the compositional distribution within the nanowires and the surrounding template.

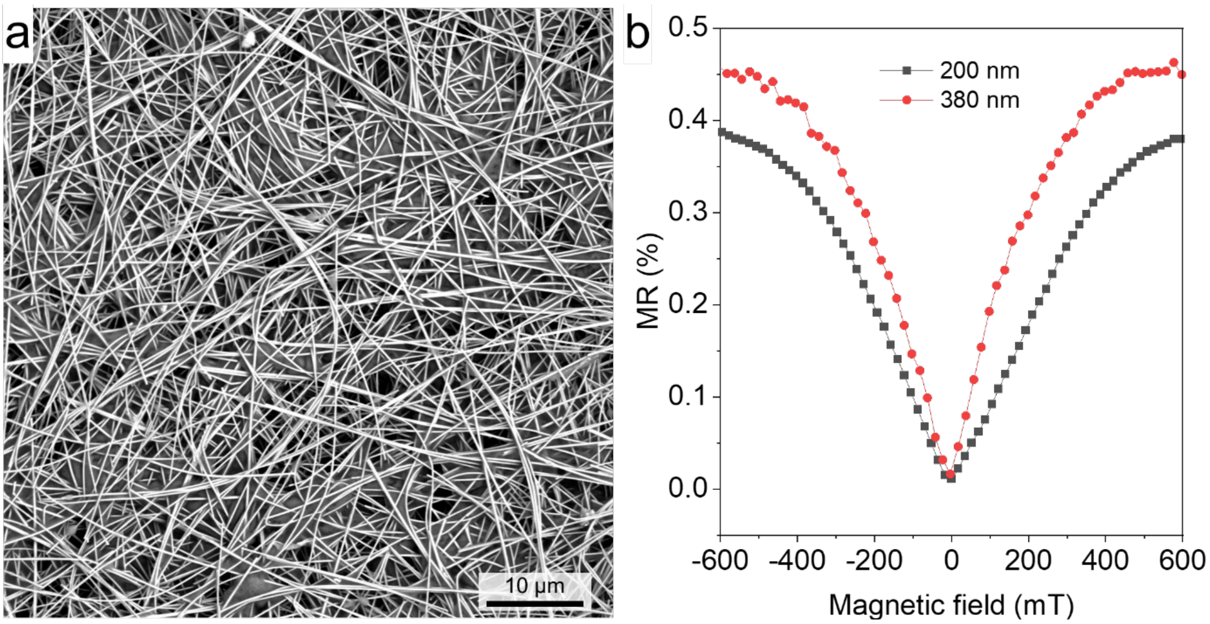

Figure S3. Influence of nanowire diameter on the MR performance. a) SEM image of CoNi nanowires with about 200-nm diameters. b) Comparison of magnetoresistance characteristics of printed sensors based on CoNi nanowires of different diameters (i.e., 200 nm, 380 nm). As the nanowire diameter decreases from 380 nm to 200 nm, the increased aspect ratio enhances shape anisotropy and easy-axis pinning, which expands the linear sensing range but slightly reduces the magnetoresistance peak value due to enhanced surface scattering and increased baseline resistance. However, it is observed that thinner nanowires (200 nm) are more prone to bending and structural deformation during the magnetic-field-guided printing process, which hinders the formation of a perfectly vertical and uniform sensor architecture. Consequently, 380 nm nanowires were selected as the optimal filler to balance superior magnetotransport properties with the necessary mechanical rigidity for high-fidelity vertical printing.

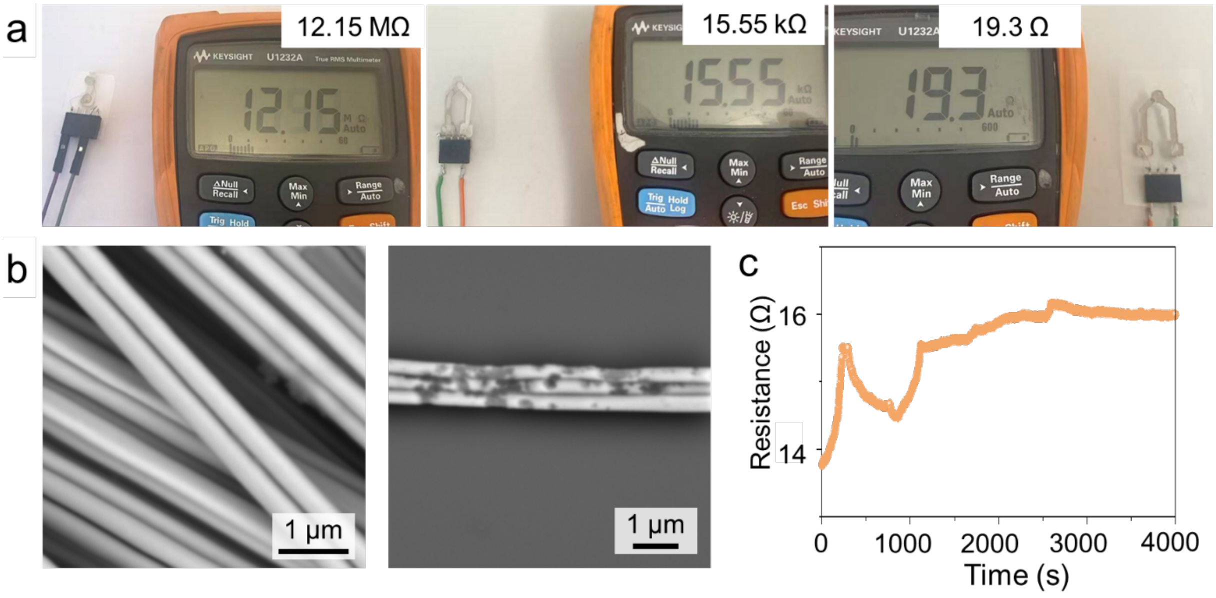

Figure S4. Printed sensors treated with phosphoric acid at different concentrations. a) Electrical resistance of printed sensors under different acid-treatment conditions. From left to right: untreated reference sensor, sensor treated with 0.005 M phosphoric acid, and sensor treated with 0.05 M phosphoric acid. b) Comparison of CoNi nanowires treated with left) 0.05 M or right) 1 M phosphoric acid. To achieve maximal sensing performance, acid treatment should optimize a practical balance between effective oxide removal and preservation of nanowire structural integrity. c) Temporal evolution of sensor resistance during acid treatment with 0.05 M phosphoric acid.

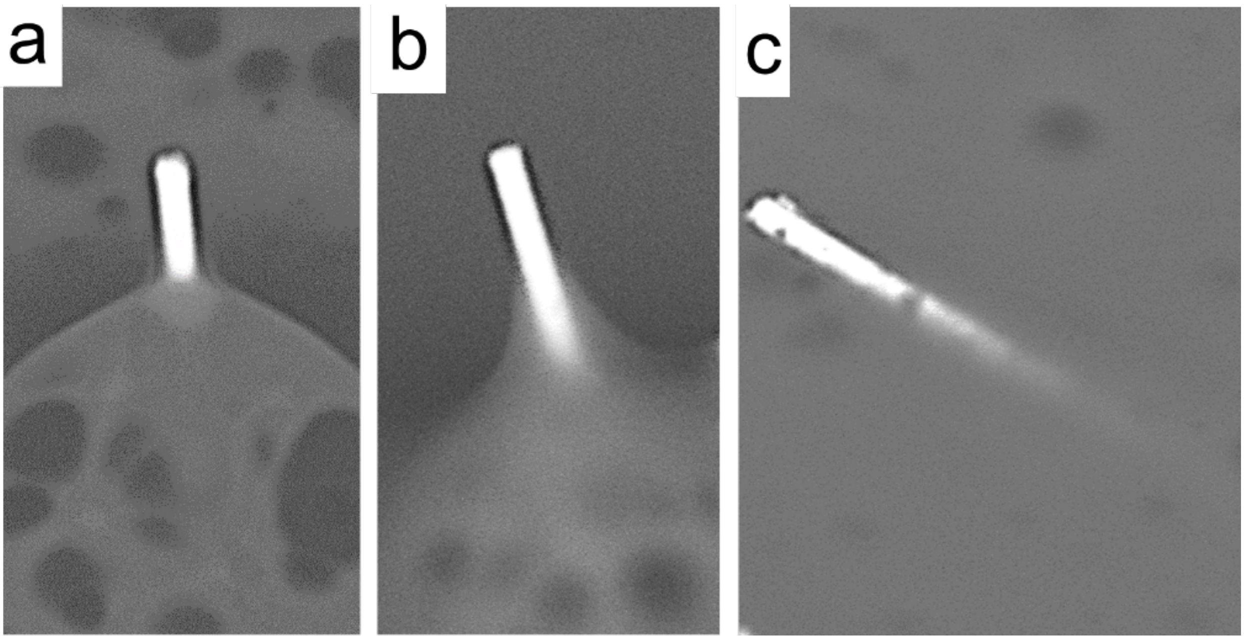

Figure S5. Representative CoNi nanowire orientation within the composite: a) vertically aligned, b) slightly tilted, c) significantly tilted configurations, validating the orientation tunability guided by external magnetic fields.

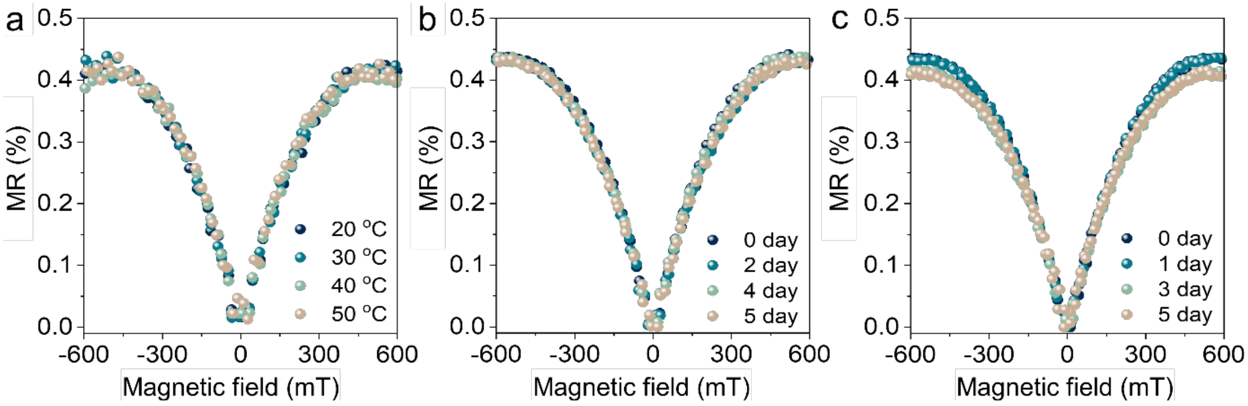

Figure S6. Magnetoresistance stability of printed CoNi-nanowire sensors under different environmental stressors: a) elevated temperatures ranging from 20 °C to 50 °C; b) immersion in water for varying durations; c) exposure to UV radiation for different periods.

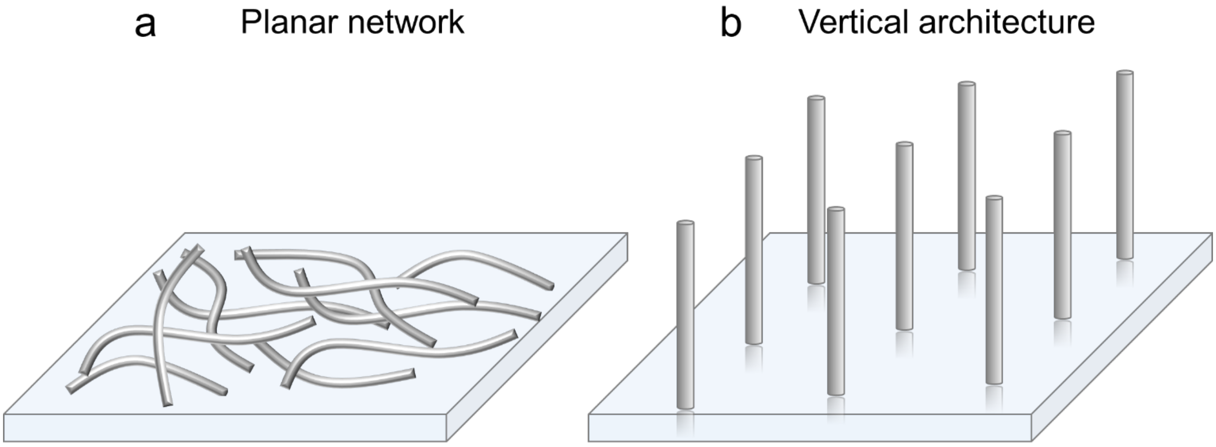

Figure S7. Schematic comparison of electron transport and optical transmission between a conventional random planar network (left) and the vertically aligned nanowire (right) architecture. In the vertically aligned architecture (right), the nanowires form direct out-of-plane bridges. This 'end-on' orientation drastically reduces the effective optical footprint of each nanowire, maximizing the aperture ratio and transparency. Furthermore, the vertical pathways decouple the electrical performance from the lateral surface coverage, overcoming the conventional 2D percolation limit.

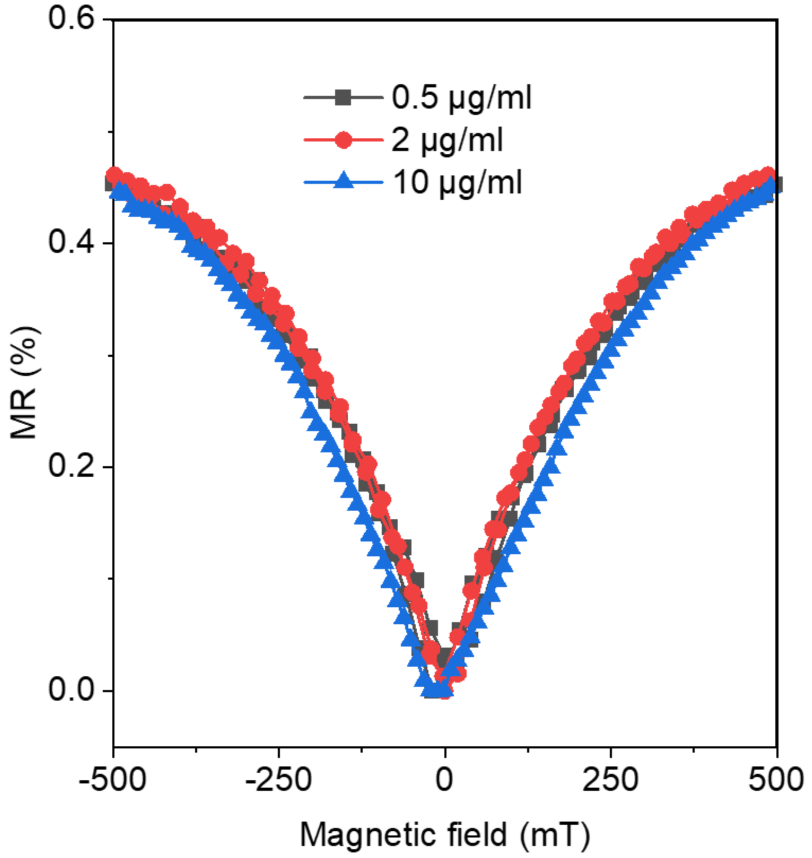

Figure S8. Magnetoresistance response of sensors printed with functional inks containing different concentrations of CoNi nanowires. In this out-of-plane configuration, the nanowires are vertically aligned to directly bridge the electrodes across the composite thickness. Consequently, charge transport is predominantly governed by the intrinsic magnetotransport properties of individual nanowires or localized vertical clusters, rather than the junction density within a lateral percolation network. This architecture ensures that the magnetoelectric performance remains remarkably consistent and independent of the nanowire loading fraction.

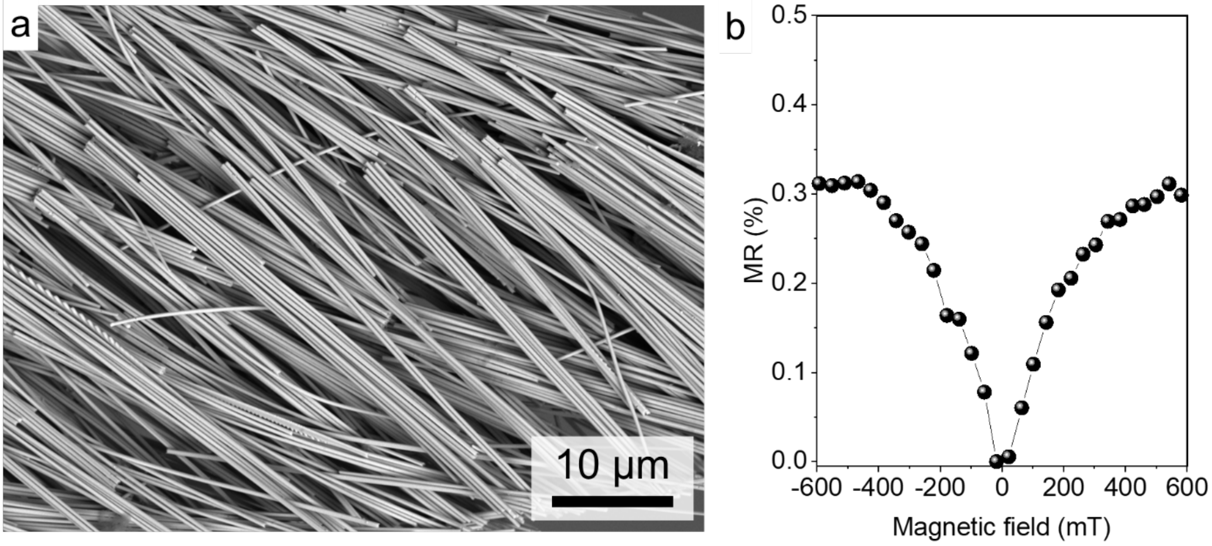

Figure S9. Printed CoNi-nanowire-based sensors with a planar architecture. a) SEM image of CoNi nanowires, forming a directionally aligned planar network. b) Magnetoresistance (MR) response of the printed sensor. Compared with the vertical configuration, the planar network-based sensor exhibits a reduced magnetoresistance response.

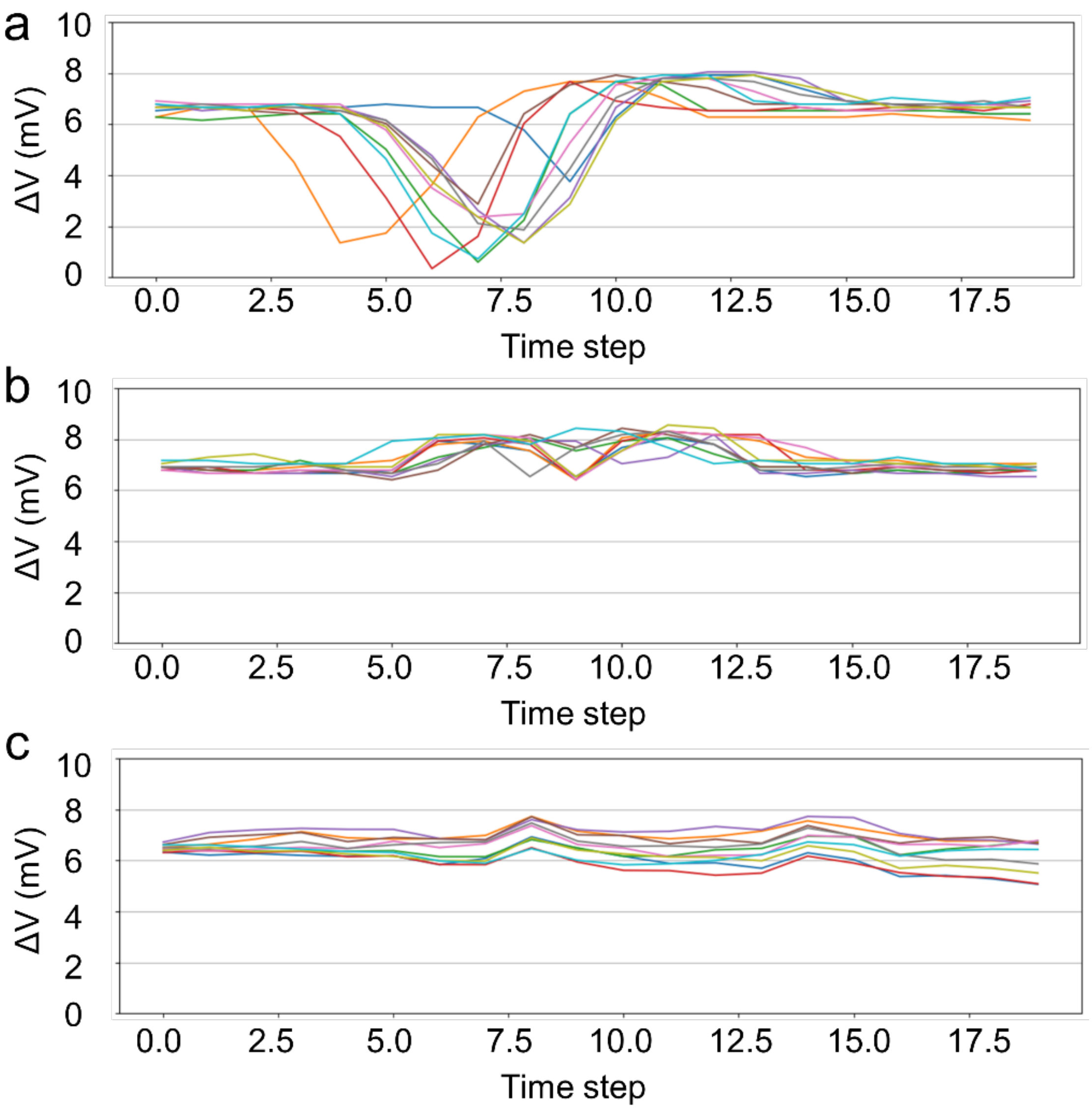

Figure S10. Representative time series signals of all three classes, selected from the training datasets. a) Signals for label "rotation". b) Signals for label "zoom". c) Signals for label "no pattern". Each labeled sample corresponds to a 20-time-step window (n = 1-20), with a sampling interval of ~0.12 s.

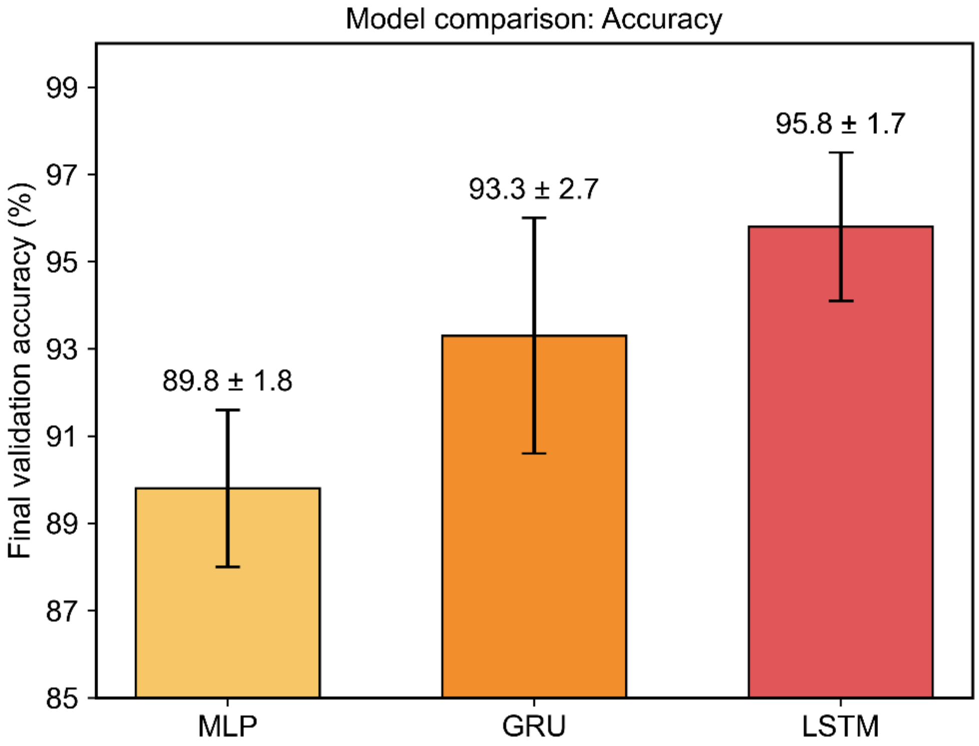

Figure S11. Final validation accuracy of MLP, GRU, and LSTM models over 10 independent training runs. Bars represent the mean final validation accuracy, and error bars indicate the standard deviation.

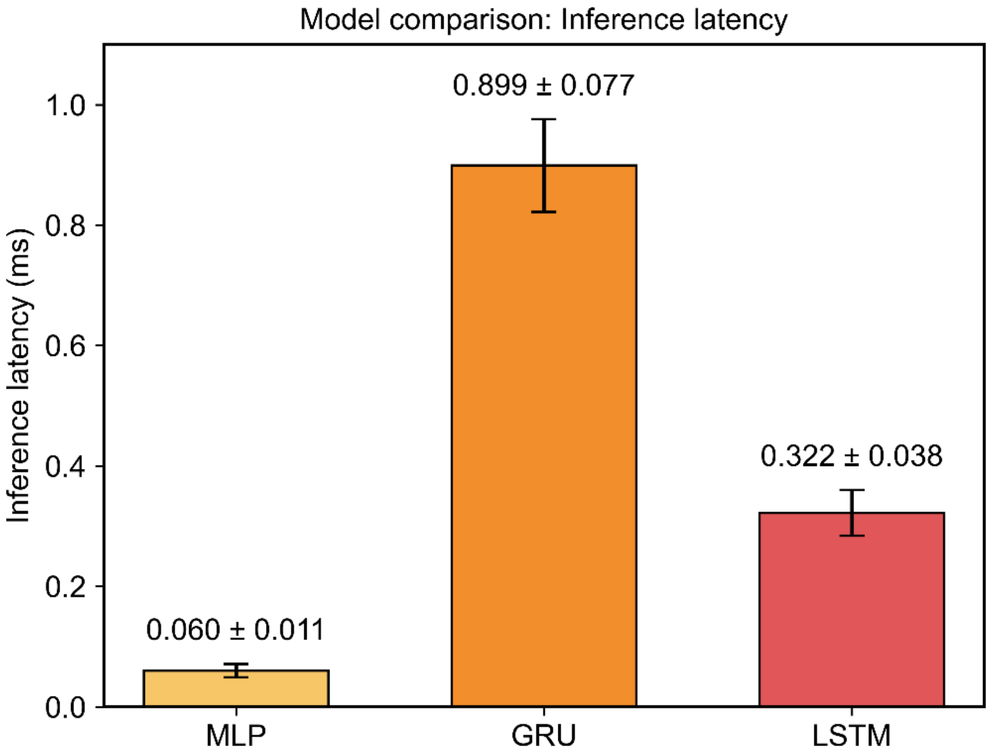

Figure S12. Model-level forward-pass inference latency of MLP, GRU, and LSTM models. For each independently trained model, the latency was measured over 300 repeated forward passes after 50 warm-up runs using a single input sequence. Bars represent the mean latency across 10 independent training runs, and error bars indicate the standard deviation.

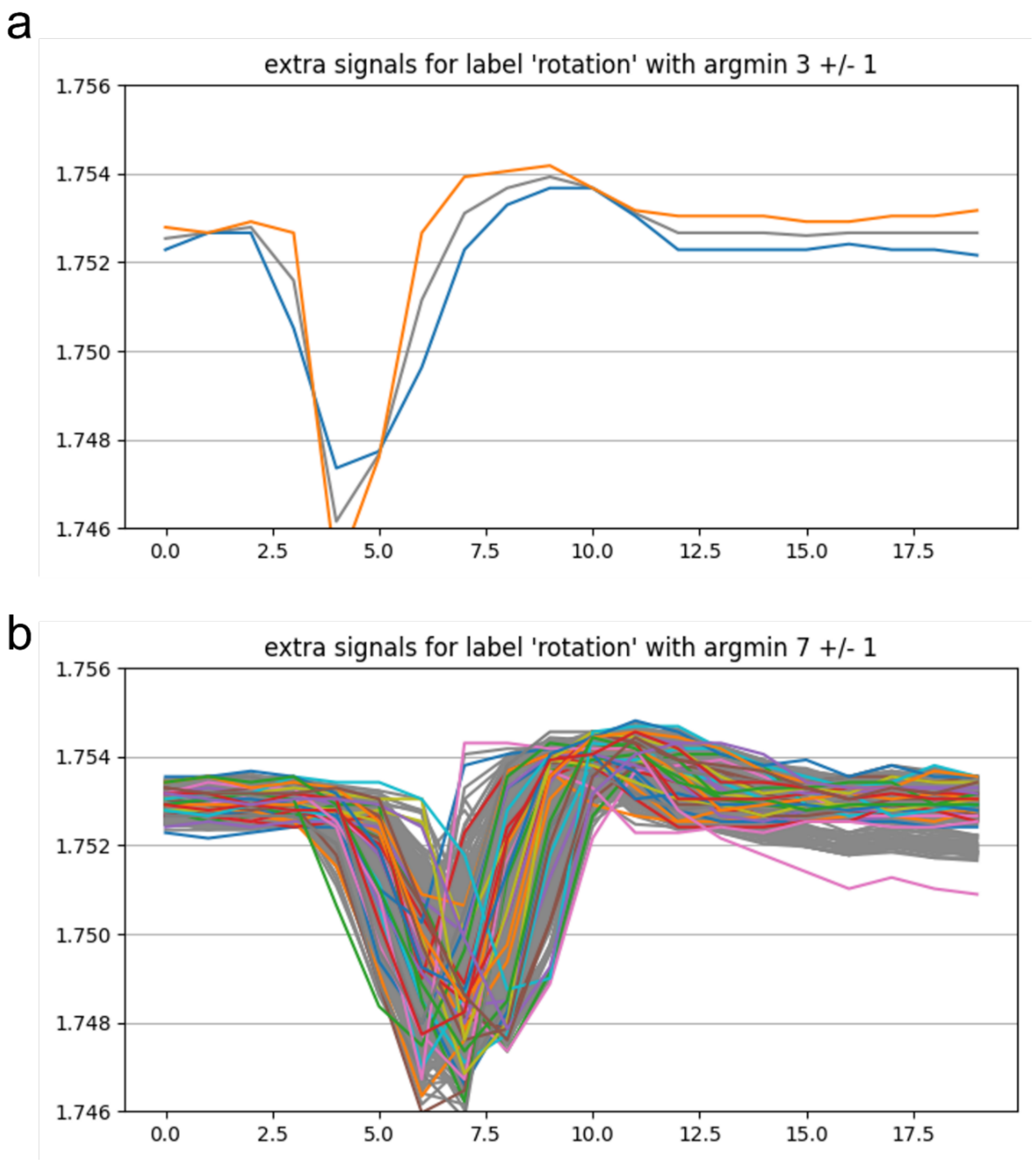

Figure S13. Illustration of the argmin-guided pairwise linear-averaging data augmentation strategy for the rotation class. For each pattern class, experimentally measured voltage sequences were grouped according to the position of their minimum voltage point, argmin(U), where U is the voltage sequence and argmin(U) represents the sampling-point index at which U reaches its minimum value. Signals with argmin(U) positions within ±1 sampling point were collected into the same augmentation subset and treated as temporally similar. New augmented samples were then generated by pairwise linear averaging of two signals within the same subset. a) Example subset centered at argmin(U) = 3, including signals with minimum positions at sampling-point indices 2–4. b) Example subset centered at argmin(U) = 7, including signals with minimum positions at sampling-point indices 6–8. Colored curves denote the original experimentally measured signals, while gray curves denote the augmented synthetic signals.

Data augmentation strategy for LSTM training

To expand the training dataset for LSTM-based temporal pattern recognition, we applied an argmin-guided pairwise linear-averaging data augmentation strategy. The purpose of this strategy was to increase the intra-class diversity of the training signals while preserving the characteristic temporal profile of each pattern class.

For each pattern class, the experimentally measured voltage sequences were first grouped according to the position of their minimum voltage point, denoted as argmin(U), where U is the voltage sequence and argmin(U) represents the sampling-point index at which U reaches its minimum value. This point corresponds to the characteristic temporal location of the pattern-specific voltage minimum and was therefore used as a simple temporal-alignment criterion. Signals with argmin(U) positions within ±1 sampling point were regarded as temporally similar and assigned to the same augmentation subset. This grouping step prevents signals with substantially shifted temporal features from being directly averaged, thereby helping preserve the class-specific waveform shape. For any two experimentally measured signals $x_i(t)$ and $x_j(t)$ within the same augmentation subset, an augmented signal was generated by pairwise linear averaging:

$$x_{aug}(t) = \frac{x_i(t) + x_j(t)}{2}$$

Only signals from the same pattern class and the same argmin-based subset were combined. Therefore, the generated signals retain the class-specific temporal pattern while introducing moderate intra-class variations in amplitude and waveform shape. This augmentation strategy increases the number of training samples without changing the original class labels. Representative examples of the original and augmented rotation signals are shown in Figure S13.

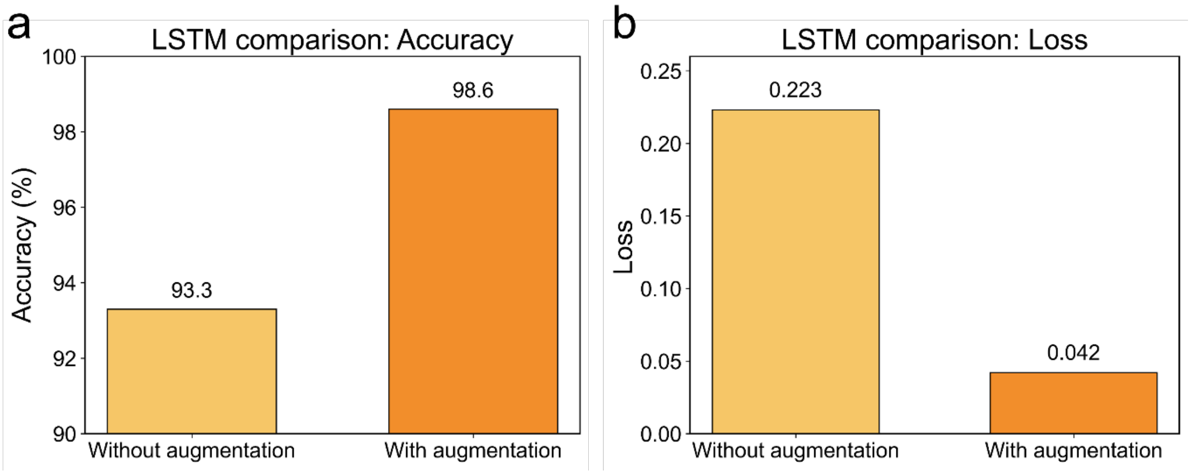

Figure S14. Effect of data augmentation on LSTM classification performance. The LSTM model was trained and evaluated with and without the argmin-guided pairwise linear-averaging data augmentation strategy. a) Final validation accuracy. b) Final validation loss. Compared with the model trained without augmentation, the model trained with augmented data increased the validation accuracy from 93.3% to 98.6% and reduced the validation loss from 0.223 to 0.042, indicating improved generalization performance on the validation data.

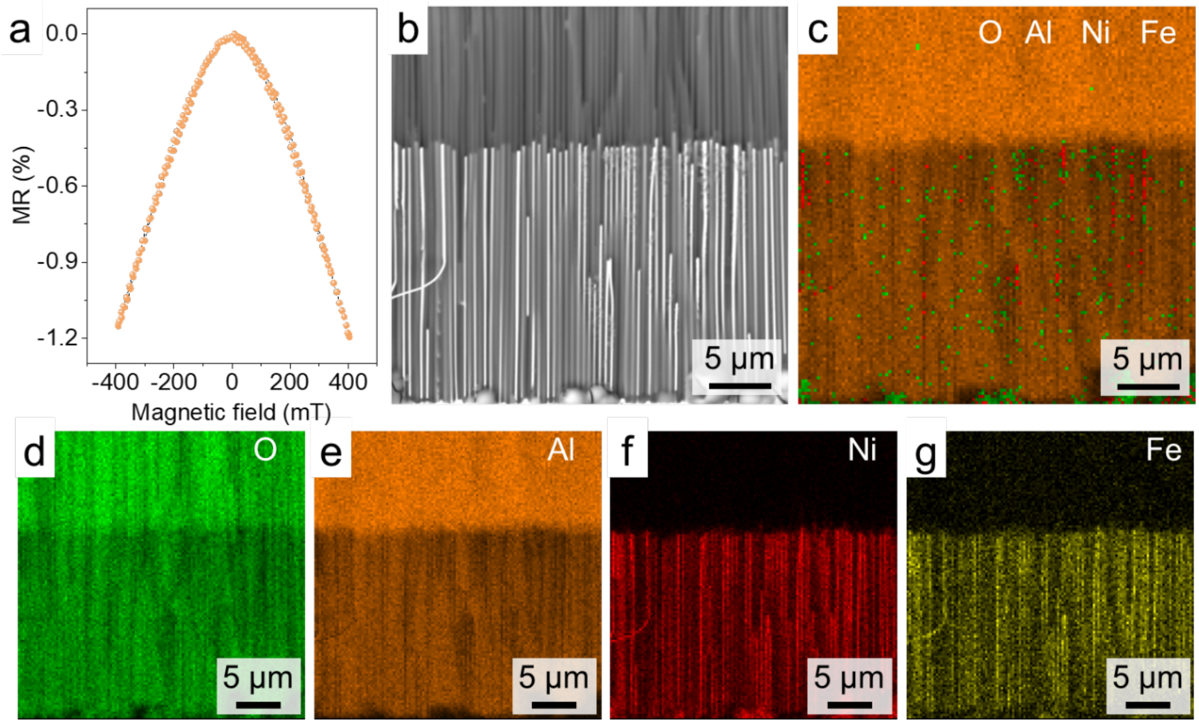

Figure S15. Printed magnetoresistive sensor based on vertically aligned NiFe nanowires. a) Magnetoresistance (MR) response of the printed sensor as a function of the external magnetic field. b) Representative cross-sectional scanning electron microscopy (SEM) image of the NiFe nanowire arrays embedded within a nanoporous anodic aluminum oxide (AAO) template, highlighting the high aspect ratio and vertical alignment. c–g) Energy-dispersive X-ray spectroscopy (EDS) elemental mapping corresponding to the region shown in panel (b), illustrating the spatial distribution and chemical homogeneity of the constituent elements within the nanowire architecture.

Table S1. Parameters exploited in three machine learning models.

Model	LSTM	GRU	MLP
Input shape	20 × 1 (Input size)	20 × 1 (Input size)	20 (Input Dim)
Hidden size	50	50	50
Hidden layer	1 LSTM layer	1 GRU layer	1 hidden linear layer
Fully connected layers	1	1	2
Output size	2	2	2
Params	10,502	7,902	1,152
Activation function	tanh, sigmoid	tanh, sigmoid	ReLU, Softmax
Optimizer	Adam	Adam	Adam
Learning rate	0.001	0.001	0.001
Loss function	CrossEntropyLoss	CrossEntropyLoss	CrossEntropyLoss
Epochs	50	50	50
Batch size	32	32	32
Train / Test split	60% / 40%	60% / 40%	60% / 40%

Table S2. Overview about training data sizes.

Data	rotation	zoom	no pattern	Total
Number of manually collected samples	50	50	-	100
Number of augmented samples	1962	2000	3485	7447
Number of samples used for training	2012	2050	3485	7547

Table S3. Evaluating the scalability from simple to more complex tasks.

Task	Number of Classes	MLP	GRU	LSTM
no pattern / rotation	2	95.0%	97.5%	97.5%
rotation / zoom	2	90.0%	92.5%	95.0%
no pattern / rotation / zoom	3	86.7%	90.0%	93.3%

Table S4. System latency and real-time performance of the LSTM-based intelligent interface.

Metric	Definition	Value
Sampling interval	Time interval between adjacent voltage samples	~0.120 s
Input sequence length	Number of voltage samples used for one LSTM prediction	20 samples
Temporal span of input window	Time span between the first and last point in one sequence, calculated as $(N - 1) \times \Delta t$	~2.28 s
Estimated median input-window waiting time	Half of the 2.28 s input-window span, assuming motion-event completion is uniformly distributed within the sequence.	~1.14 s
Sliding-window step	Number of samples by which the input window was shifted in the real-time demonstration	5 samples (~ 0.60 s)
Classification evaluation interval	Time interval between two consecutive LSTM classifications, calculated as sliding-window step × sampling interval	~ 0.60 s
Preprocessing latency	Standardization, reshaping, and tensor conversion	0.208 ± 0.042 ms
Pipeline LSTM inference latency	LSTM forward-pass latency measured within the complete processing pipeline	0.435 ± 0.078 ms
Command mapping latency	Argmax classification and label-to-command assignment	0.017 ± 0.005 ms
Total algorithmic latency	Preprocessing + pipeline LSTM inference + command mapping	0.660 ± 0.117 ms
Pipeline LSTM inference speed	Prediction speed based on pipeline LSTM inference only	~2300 predictions s^{-1}
Total algorithmic processing speed	Prediction speed based on preprocessing + inference + command mapping	~1515 predictions s^{-1}
